# Association of brain-derived neurotrophic factor levels and depressive symptoms in young adults with acne vulgaris

**DOI:** 10.1186/s12888-019-2182-8

**Published:** 2019-06-24

**Authors:** Hong-yi He, Jin-lan Tian, Yong-qiong Deng, Xia Xiong, Yang Xu, Yong-mei Liao, Jing Fang, Xia Feng, Xin Ye, Chang-qiang Li

**Affiliations:** Department of Dermatology, The Affiliated Hospital of Southwest Medical University, Luzhou, Sichuan 646000 People’s Republic of China

**Keywords:** Acne vulgaris, Serum BDNF levels, Depression

## Abstract

**Background:**

Brain-derived neurotrophic factor (BDNF) is one of the proteins that contributes to the survival, growth, maintenance of neurons, and plays important roles in the pathophysiology of depression. It has been reported that depression is closely associated with the pathogenesis of acne vulgaris disease. But, there is no report of serum BDNF levels in patients with acne vulgaris. The study aimed to determine the potential association between BDNF and depressive symptoms in young adults with acne vulgaris.

**Methods:**

In this analytical cross-sectional study, the serum BDNF levels were measured in peripheral blood samples of 20 consecutive acne vulgaris patients with depression and 98 consecutive acne vulgaris patients without depression and also compared it with a 59 healthy control group by using a ELISA. The potential correlation between the BDNF levels, interleukin-6 (IL-6), tumour necrosis factor-alpha (TNF-α), and depressive symptoms such as nine-item patient health questionnaire (PHQ-9) and Athens insomnia scale (AIS) were evaluated with multivariate logistic regression analysis.

**Results:**

Our results showed that levels of BDNF expression were lower in consecutive acne vulgaris patients when compared with healthy control (*P <* 0.05). There was a negative correlation between levels of BDNF and the PHQ-9 scores (r = − 0.486, *P <* 0.001). Furthermore, acne vulgaris patients with depression showed lower serum BDNF levels (10.96 ± 2.12 ng/ml) compared with acne vulgaris patients without depression (13.85 ± 2.47 ng/ml), as well as with healthy control (14.35 ± 2.70 ng/mg; both *P* < 0.05). No difference was found in serum BDNF levels between healthy control and acne vulgaris patients without depressive symptoms (z = 0.964, *P* > 0.05). Similarly, the overall area under the curve of receiver operating characteristic was 0.82, indicating the highly conserving of serum BDNF levels as an biomarker for screening of depression in young adults with acne vulgaris (72% sensitivity and 85% specificity).

**Conclusion:**

Serum BDNF levels were decreased and negatively associated with depressive symptoms in young Chinese adults with acne vulgaris.

**Electronic supplementary material:**

The online version of this article (10.1186/s12888-019-2182-8) contains supplementary material, which is available to authorized users.

## Background

Acne is a chronic inflammatory disease of the pilosebaceous unit, which consists of open comedones (blackheads), closed comedones (whiteheads), nodules, pustules, and Papule*s. acne* has a prevalence of the skin in approximately 86% of adolescents [1–4]. Young adults with acne vulgaris have a greater chance of depression, with 3.02% in Chinese’s acne vulgaris and 4.45% in Americana’s acne vulgaris [5, 6]. However, previous studies have reported that depression was identified in approximately 68.3% of acne vulgaris patients [7, 8]. Depression in acne vulgaris patients is assessed primarily using clinical features and some depression scales [9]. In actual clinical practice, diagnostic accuracy of acne vulgaris is highly variable, and result is often affected by the type of depression scale, the patient’s education level, and personal views of patient [10]. Clinically, isotretinoin is the first-line treatment of patients with moderate-to-severe acne vulgaris [11, 12]. Certainly, finding the non-invasive scanning biomarkers and their incorporation into clinical practice could alter the future outcomes of acne vulgaris treatment [13]. Therefore, the development of novel biomarkers with high sensitivity and specificity for scarring the depression in acne vulgaris patients are still needed.

Brain-derived neurotrophic factor (BDNF) is a group of neurotrophin family of growth factors, which plays a critical role in many important biological processes such as survival, proliferation, and maintenance of neurons [14]. In the normal cells, BDNF as a certain neuron helps to support the survival of existing neurons in both central and peripheral nervous system, and resulting the growth and differentiation of new neurons and synapses [15, 16]. Our literature reviews implied that BDNF have been associated with pathophysiology of depression in patients with poststroke depression [17], systemic lupus erythematosus [18], multiple sclerosis [19], and postpartum depression [20]. BDNF abnormalities could contribute to the dysfunction of astrocytes and microglia in depression circuits [21–23]. In patients with depression, BDNF was down-regulated in the hippocampus and medial prefrontal cortex; which are closely related to emotional and cognitive functions [22, 24]. Low concentrations of serum BDNF in antidepressant-free patients is a reliable biomarker for quality of life variations [23, 25, 26]. Although many studies have shown that depression roles in acne vulgaris, there is no comprehensive study on BDNF levels in acne vulgaris patients with depression [27]. Thus, understanding the mechanisms of BDNF has considered as a promising scarring approaches of the acne vulgaris therapy [25].

Therefore, we aimed to evaluate the serum BDNF levels in the pathogenesis of acne vulgaris patients with depression. We evaluated the serum BDNF levels in acne vulgaris patients with and without depression and compared with age-matched healthy control subjects. Furthermore, we planned to document the evidence for the use of BDNF as a scarring biomarker to predict other clinical pathological feature outcomes of acne vulgaris.

## Methods

### Subjects and study design

This analytical cross-sectional study was carried out during a 6-month period from Jan. 2017 to Jun. 2017. The participants were adult patients who were referred to dermatology department of Affiliated Hospital of Southwest Medical University in Luzhou, Sichuan, China. Totally, 180 patients were selected for study based on inclusion/exclusion criteria (18–24 yrs.). All of the participants were free of heavy alcoholic drinking and smoking. The participants were into three groups; 20 acne vulgaris patients with depression, 98 patients without depression, and 59 healthy control. The participants excluded if patients or healthy individuals with history of known mental retardation; patients with some somatic diseases such as heart, pulmonary diseases, and diabetes that affect their mental status; and patients who used topical or systemic medicines disposing acne during 1 month before refer for acne. In additions, histories of acne vulgaris, and/or participation in simultaneous clinical trials were considered as a main exclusion criterion.

### Enrollment and assessment criteria

Age, gender, occupation, family annual income (high family annual income was defined as annual per capita household income>40.000 yuan), disease duration, family history of acne vulgaris, and history treatment with oral antibiotics for more than 2 weeks or with isotretinoin for more than 4 weeks in past 3 months were recorded through a questionnaire survey. The severity of acne vulgaris was evaluated using the Global Acne Grading System (GAGS) [28]. The sleep quality of patients were assessed using Athens Insomnia Scale (AIS), which is a self-administered psychometric instrument [29]. Dermatology Life Quality Index (DLQI) was applied to assess patients’ quality of life [30]. It is a scale used specifically for dermatology, and is composed of 10 questions that fall under 6 categories. A higher total score indicates a lower quality of life. The primary outcome variable of depressive symptoms were scored according the nine-item patient health questionnaire (PHQ-9), which is a depression screening scale based on the criteria from Diagnostic and Statistical Manual of Mental Disorders-IV (DSM-IV) [31, 32]. In our study, participants with a PHQ-9 score > 10 were categorized as depressive symptoms [33].

### Laboratory tests

Fresh blood samples from all acne vulgaris patients and healthy persons were collected by vacuum tubes on an empty stomach in the morning (before 9.30 AM). All participants were non-fasting in during the sampling. Then, the blood sample coagulated at room temperature and centrifuged at 3000 rpm. All serum samples were collected and then stored for less than 6 months at − 20 °C [34]. If precipitation occurs again during storage, centrifugation was taken once again. The measurement of BDNF was performed by ELISA according to the manufacturer’s instructions (Bluegene Biotech Co., Shanghai, China). The content of interleukin-6 (IL-6) and tumour necrosis factor-alpha (TNF-α) in serum were determined by radioimmunoassay according to the manufacturer’s instructions (Northern Institute of Biotech., Beijing, China). The intra- and inter-assay coefficients of variation of IL-6 were less than 7 and 15%, respectively. Also, the intra- and inter-assay coefficients of variation of TNF-α were less than 10 and 15%, respectively.

#### Statistical analyses

All quantitative data were transferred to excel and the statistical analyses were computed with SPSS software for Windows (Version 21, SPSS Inc., Chicago, Illinois, USA). Data were presented as means ± Std. Deviation (SD) or median (range). Analysis of variance (ANOVA) was used for three-group continuous data and Student-Newman-Keuls test was used for Post-Hoc analyses. Spearman’s Rank correlation was used for bivariate correlations. The relation of serum BDNF with depression was investigated using logistic regression models. Further, receiver operating characteristic curves (ROC) was used to test the overall diagnostic accuracy of BDNF. For all tests, *P <* 0.05 considered statistically significant. All charts were designed by Prism 5.0 (GraphPad, La Jolla, CA, USA).

## Results

### Background of study population

The demographic and clinical characteristics of the subjects were compared in Table 1. There was no difference between the groups in terms of age, gender, and family history of acne vulgaris (*P >* 0.05). In this study, 118 acne vulgaris patients (69% women, 20.97 ± 1.54 age) and 59 healthy subject (61% women, 21.02 ± 1.50 years) were studied. Summarized data from all individuals showed that the quantity of serum levels of BDNF in acne vulgaris patients were significantly lower than that in healthy controls (13.35 ± 2.65 vs. 14.35 ± 2.70; *P* = 0.038). Median acne vulgaris duration was 4.86 ± 2.44 year**s** and GAGS score was 20.19 ± 6.92. In this study, the mean PHQ-9 score was 6.17 ± 4.55 in acne vulgaris patients and 3.44 ± 2.17 in healthy controls group. According to PHQ-9 score, depression was identified in 17% of acne vulgaris patients. As expected, these data suggest that quality of life in patients with acne vulgaris are critically lower than healthy individuals. In line with previous studies [35], IL-6 and TNF-α frequency showed no difference in the acne vulgaris patients compared with healthy controls (*P ≤* 0.05; Table 1).

**Table 1:** Socio-demographic characteristics of acne vulgaris patients and healthy controls

	Acne vulgaris patients (*n =* 118)	Healthy controls (*n =* 59)	*P-value* ^a^
*Demographic variables (n (%))*			
Age (*yrs.*)	20.97 ± 1.54	21.02 ± 1.50	0.746
Gander (*f/m*)	68/50	23/36	0.312
Drop out and unemployed	21 (18)	13 (22)	0.50
High family annual income	69 (58)	42 (71)	0.099
Family history of acne vulgaris	26 (22)	11 (19)	0.601
Disease durations (*yrs.*)	4.86 ± 2.44	–	–
*Medication variables (%)*			
Oral isotretinoin	42.37	–	–
Oral antibiotics	38.14	–	–
*Quality of life variables*			
PHQ-9	6.17 ± 4.55	3.44 ± 2.17	< 0.001
AIS	5.66 ± 3.49	4.03 ± 1.90	0.002
GAGS	20.19 ± 6.92	–	–
DLQI	5.83 ± 4.69	–	–
*Laboratory variables*			
BDNF (*ng/ml*)	13.35 ± 2.65	14.35 ± 2.70	0.038
IL-6 (*pg/ml*)	36.43 ± 10.14	36.40 ± 11.55	0.681
TNF- α (*pg/ml*)	455.28 ± 224.13	418.82 ± 201.14	0.256

All data expressed as mean ± SD (range) of the mean of individual groups

^a^ Mann-whitney U test and chi-square test were used

Abbreviation: *IQR* interquartile range, PHQ-9:9-item Patient Health Questionnaire, *AIS* Athens Insomnia Scale, *GAGS* Global Acne Grading System, *DLQI* Dermatology Life Quality Index, *BDNF* brain-derived neurotrophic factor, *IL-6* interleukin-6, *TNF-α* tumor necrosis factor-alpha, ^a^ Mann–Whitney U test and chi-square test were used

### Serum levels of BDNF was inversely correlated with depression

**Fig. 1:**
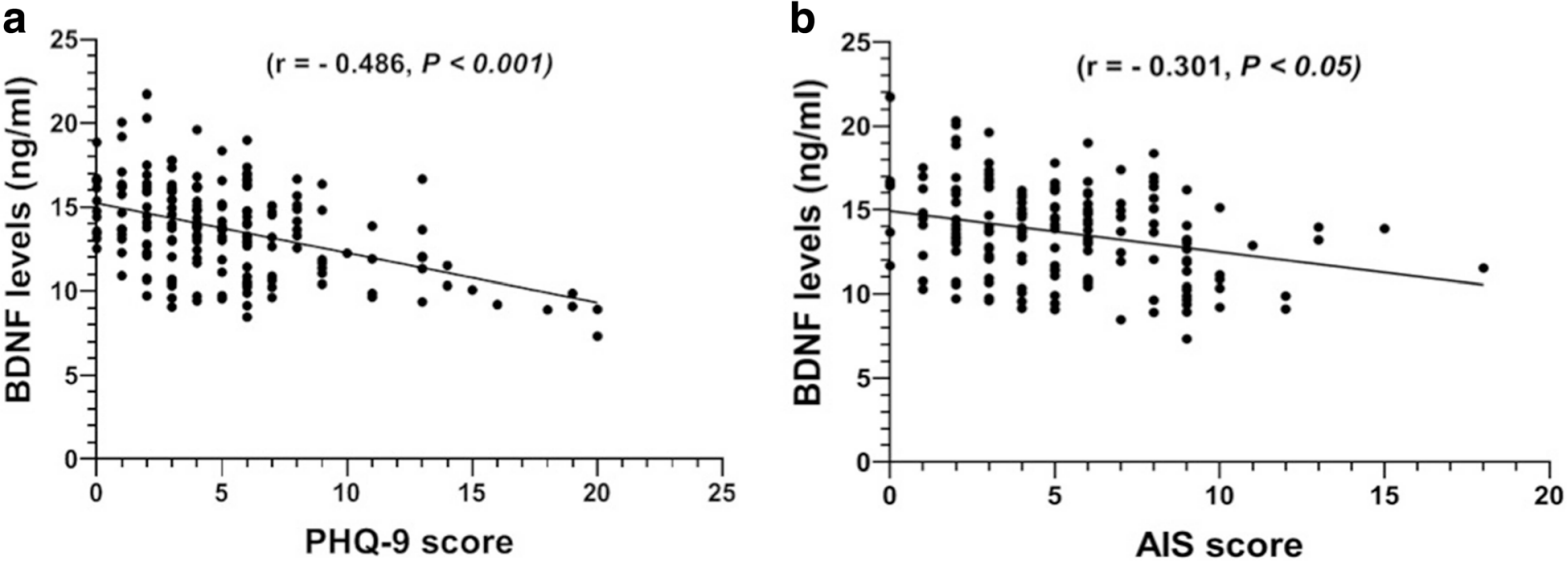
Correlations of serum levels of BDNF with PHQ-9 score (**a**) and with AIS score (**b**) in acne vulgaris patients. The line is the regression line calculated with SPSS. Data were analyzed by Spearman’s rank correlation coefficient (r). BDNF, brain-derived neurotrophic factor; PHQ-9, 9-item Patient Health Questionnaire; and AIS, Athens Insomnia Scale

Our results clearly show a negative correlation between serum levels of BDNF and PHQ-9 score in acne vulgaris patients (r = − 0.486, *P* < 0.001; Fig. 1a). In addition, BDNF levels also showed a negative correlation with AIS score (r = − 0.301, *P* < 0.001; Fig. 1b). In general, lower serum levels of BDNF are correlated with depression severity in patients with acne vulgaris. Besides, we found no significant correlation between BDNF levels and other depression parameters such as, GAGS score and DLQI score as well as levels of IL-6 and TNF-α (*P >* 0.05; Additional file 1: Table S1).

### BDNF decreased in acne vulgaris patients with depression

**Fig. 2:**
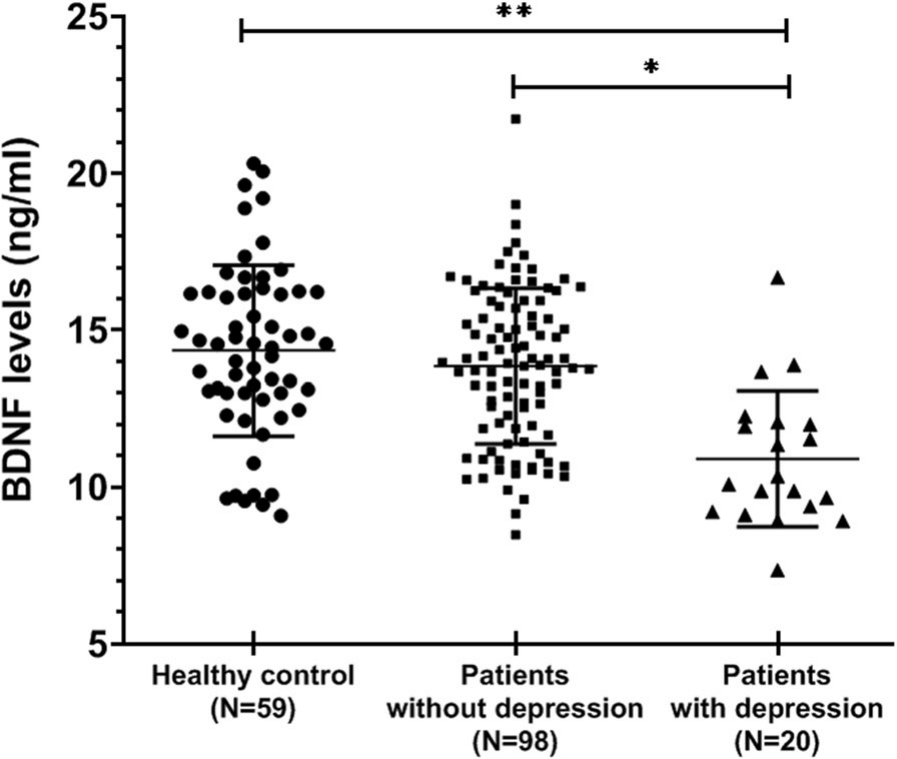
Comparison of the serum levels of BDNF in acne vulgaris patients with depression (*N* = 20), acne vulgaris patients without depression (*N* = 98), and healthy control (*N* = 59). In general, lower percentages of BDNF were observed in acne vulgaris patients with depression. Horizontal bars show mean ± SD; BDNF, brain-derived neurotrophic factor (*P < 0.05, ** *P <* 0.001)

Magnitude of depression and BDNF between the acne vulgaris patients with depression, acne vulgaris patients without depression and healthy controls group are shown in Fig. 2. The serum BDNF levels were markedly decreased in patients with depression compared with patients without depression and healthy cases (10.96 ± 2.12 vs. 13.85 ± 2.47, 10.96 ± 2.12 vs. 14.35 ± 2.70, respectively; *P ≤* 0.05). But we didn’t observe any significant differences between acne vulgaris patients without depression and controls (13.85 ± 2.47 and 14.35 ± 2.70 ng/ml, respectively; *P >* 0.05).

### BDNF associated with depression among acne vulgaris patients

To evolution whether the serum levels of BDNF are associated with depression in acne vulgaris patients, we conducted the multivariate logistic regression analysis. We found out statistically significant associations between dependent and independent variables. Serum levels of BDNF were independently associated with depression in acne vulgaris patients with depression. In univariate analysis, acne vulgaris patients with depression had lower family annual income, higher probability of family history of acne vulgaris, dropping out from school and currently unemployed, higher AIS score, higher DLQI score, and lower BDNF levels. Furthermore, there was an increased risk of depression associated with lower serum BDNF levels (OR: 0.52, 95% CI: 0.35–0.76; *P* < 0.05). Furthermore, lower AIS score, dropping out from school, and currently unemployed, and lower family annual income were also predictors of depression in acne vulgaris patients (Table 2).

**Table 2:** Characteristics of acne vulgaris patients stratified according the depression

	Patients without depression (*n =* 98)	Patients with depression (*n =* 20)	*P-value* ^a^	OR (95%CI)	*P-vlaue*’
*Demographic variables (n (%))*					
Age (*yrs.*)	20.98 ± 1.57	20.95 ± 1.36	0.99	1.12 (0.66–1.90)	0.743
Gender (*f/m*)	40/48	11/9	0.149	1.33 (0.24–7.45)	0.743
Graduate with no work (*n*)	14 (14)	7 (35)	0.027	11.05 (1.67–73.12)	0.013
High family annual income (*n*)	64 (65)	5 (25)	0.001	7.71 (1.69–35.21)	0.008
Family history of acne vulgaris (*n*)	18 (18)	8 (40)	0.033	0.82 (0.12–5.43)	0.837
Disease durations (*yrs.*)	4 (3–6)	5.5 (4–7)	0.267	0.96 (0.67–1.38)	0.839
*Medication variables*					
Oral isotretinoin	40.82	55	0.210	0.64 (0.08–4.83)	0.663
Oral antibiotics	36.73	45	0.488	1.18 (0.19–7.23)	0.857
*Quality of life variables*					
AIS	4.96 ± 3.06	9.10 ± 3.39	0.000	1.52 (1.17–1.98)	0.002
GAGS	19.85 ± 6.85	21.85 ± 6.98	0.297	1.01 (0.85–1.19)	0.920
DLQI	5.28 ± 4.34	8.55 ± 5.32	0.01	1.06 (0.88–1.28)	0.558
*Laboratory findings*					
BDNF (*ng/ml*)	13.85 ± 2.47	10.96 ± 2.12	0.000	0.52 (0.35–0.76)	0.001
IL-6 (*pg/ml*)	36.18 ± 10.36	37.63 ± 8.89	0.247	1.02 (0.94–1.11)	0.671
TNF- α (*pg/ml*)	453.40 ± 232.49	464.55 ± 177.27	0.493	1.00 (0.99–1.01)	0.830

All data expressed as mean ± SD (range) of the mean of individual groups

^a^ Mann-whitney U test and chi-square test were used

^*b*^ Binary logistic regression was used

Abbreviation: *IQR* interquartile range, PHQ-9:9-item Patient Health Questionnaire, *AIS* Athens Insomnia Scale, *GAGS* Global Acne Grading System, *DLQI* Dermatology Life Quality Index, *BDNF* brain-derived neurotrophic factor, *IL-6* interleukin-6, *TNF-α* tumor necrosis factor-alpha

### BDNF as a predictor for evaluating depression in acne vulgaris patients

**Fig. 3:**
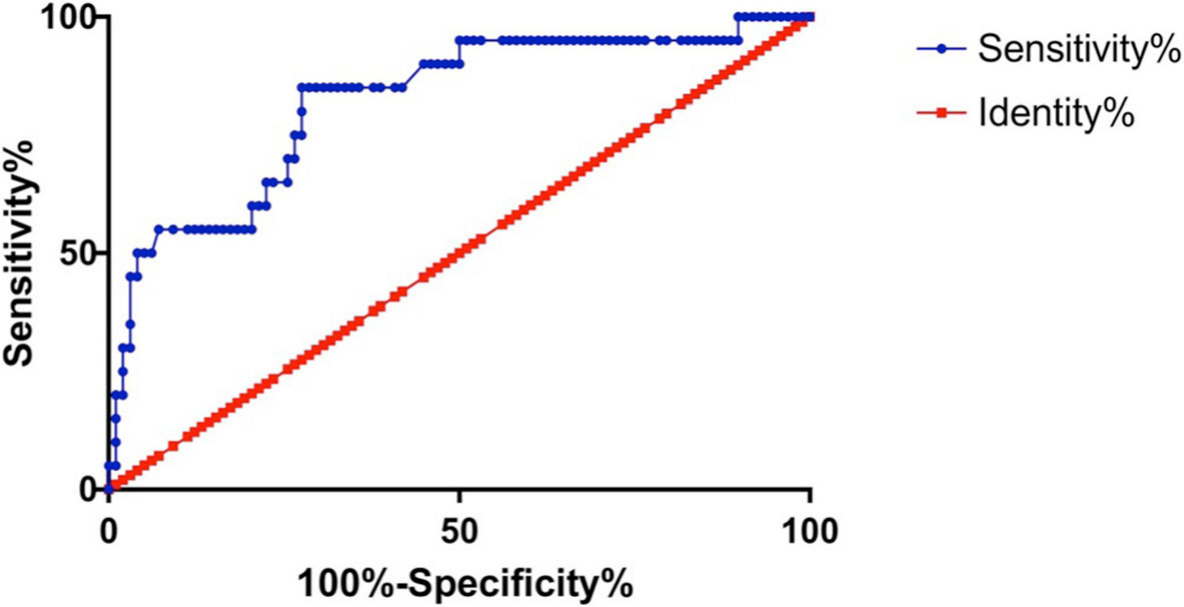
Receiver operating characteristic curve for BDNF. As a ROC curve shows, the 12.28 ng/ml of BDNF cab be indicator for screening of depression in young adults with acne vulgaris with 72% sensitivity and 85% specificity

In this study the prediction capacity of depression in acne vulgaris patients was assessed using ROC (Fig. 3). Based on the ROC curve, overall area under the curve (AUC) was 0.82 (95% CI: 0.71–0.93; *P <* 0.001). The optimal cut-off value of serum BDNF levels as an indicator for screening of depression was estimated to be 12.28 ng/ml, which yielded a sensitivity of 72%, specificity of 85%, a true positive rate of 72%, and a false positive of 15%.

## Discussion

In this report, we present the first detailed analysis of BDNF levels and depression in young Chinese acne vulgaris patients. Our findings indicate a significant decrease of BDNF in the acne vulgaris patients with depression in compare with control subjects. Moreover, BDNF negatively associated with depression among acne vulgaris patients.

Depression is commonly encountered in acne vulgaris patients. Previous studies had reported that depression was identified in 8–29% of acne patients [5–8]. Similarly, in our study, 17.7% of acne patients were documented with depression. The median PHQ-9 scores in acne vulgaris patients were higher than control group (*P <* 0.001). Some studies have attempted to explore causes of increased rates of depression in acne patients [7, 36]. Yang YC. et al. (2014) reported that gender and acne are independently associated with acute depression [37]. Acne-specific quality of life is associated with depression in patients with acne, and they also indicated that there was no correlation between acne severity and depression [9, 36]. However, in a recent study, a positive correlation was found between depression and acne severity [7, 37]. In our study, we use a logistic regression model to explore independent risk factors of depression in patients with acne. In line with Yazici K. et al. (2004) the severity of disease is not related to depression in acne patients [8]. Furthermore, no any significant correlation was reported between depression with gender, quality of life, and isotretinoin therapy [38, 39]. Poor AIS score, lower serum levels of BDNF, dropping out of school, currently unemployed, and lower family annual income were independent risk factors for depressive symptoms after adjustment by variables [39]. The differences in results across these studies may be related to the differences in study population, sample size, scales, and statistical methods used in these studies. Serum BDNF concentrations show strong seasonal variation. According to a coherent pattern, the serum BDNF concentrations increased in the spring-summer period and decreased in the autumn-winter period [23, 34, 40, 41]. Furthermore, serum levels of BDNF strongly correlated with time of blood withdrawal, storage, urbanity, age, and sex [34, 40]. So, all sampling of this study was done in the early morning of spring-summer period in non-fast cases [41].

Neurotrophic factor hypothesis is one of the pathogeneses of depression. Undoubtedly, depression requires long-term treatment for achieve best clinical efficacy. The results of brain imaging studies demonstrate that depression effects the specific brain regions, such as limbic brain regions. For example, volume of the hippocampus regions in patients with moderate to severe depression have remarkably smaller than healthy control [42]. Recently studies tried to analysis the BDNF function in depression mechanism, by considering the important effect of BDNF in promoting the survival and differentiation of neurons, as well as enhancing dendritic spines complexity and synapses plasticity [43–45]. The BDNF as an activity-dependent neuronal plasticity, by reduction of tropomycin receptor kinase (Trk)-B (TrkB) as specific neurotrophin receptors, increased the depression symptoms. Genetic BDNF knock-in and knock-out models shows that activations of Trk were reduced in BDNF-deficient mice [25, 45]. These alternations of the BDNF and TrkB levels in serum may be useful to introduced as a non-invasive biomarker for clinical improvement of depressive symptoms [45]. A reduced levels of brain BDNF was observed in subjects with depressive symptoms, while can be reversed by giving different antidepressant treatments [46, 47]. Some studies reports that rodent’s behavioral response to a forced swim stressor does not reflected depression [43, 44]. However, depression-like behavior in the studies listed above was also assessed by sucrose preference test and open field test. A reduced levels of brain BDNF was also observed as peripheral manifestations in patients with of depression [26]. There are also several reports demonstrating that expression of BDNF in the hippocampus was decreased during chronic depression and normalizes during remission [23, 26, 48]. Moreover, compared with subjects who were not treated with antidepressants, an increased BDNF expression was found in the hippocampus in subjects treated with antidepressant medications [49]. The link between BDNF and depression is also confirmed at the genetic level. Some studies demonstrated that single nucleotide polymorphism Val66Met in the BDNF gene was associated with decreased hippocampal volume in persons with major depressive disorder (MMD) and may contribute to a genetic predisposition for depression [50, 51].

In addition to the central nervous system, BDNF was also found in peripheral blood, which was mainly stored in the platelets of α-particles and released from platelets to plasma or serum upon agonist stimulation [52, 53]. BDNF triggers the peripheral immunity and an immunotrophic function in MMD [54, 55]. Patas K. et al., (2014) introduce the BDNF as a novel and cytokine-specific screening biomarker in immune-based antidepressive therapy [54]. They report that BDNF levels are positively associated with IL-6 levels in the pathophysiology of MMD [54]. Some scientists compered the BDNF levels in the serum and brains. They found that there was a positive correlation between BDNF levels in brain tissue and serum [56]. This suggested that changes in serum BDNF levels can reflect the changes of brain BDNF levels and may be associated with the pathophysiology of depression. BDNF released from platelets could also be increased by various types of antidepressive therapies [57]. A compared with healthy control, the serum BNDF levels of acne vulgaris patients with chronic depression was decreased, and can be higher through antidepressant treatment [48]. Consistent with previous studies, in our research, acne vulgaris patients showed lower serum BDNF levels compared with healthy controls [26, 48]. Some other investigations suggest that low serum levels of BDNF are an abnormality index that is distinct during depression and normalizes during remission [23, 48]. Incensing levels of BDNF during antidepressant treatment are not parallel with severity of depressive symptoms. Therefore, further longitudinal studies are needed to better understand the role of BDNF in development of depression in MMD [26, 48].

We should point out that there are some limitations in this investigation. First, this is a cross-sectional study, so it cannot determine a cause-and-effect relationship between our independent variables and depressive symptoms. Second, we chose a PHQ-9 score of 10 as a cut-off to define depression, and the lack of clinical interviews to confirm the syndrome of depression mandates that results be interpreted with caution. Third, to better assess the relationship between isotretinoin and depression, the patients chosen in the study were aged 18–24 years, which is not the age group most susceptible to acne vulgaris. Certainly, well-designed functional studies in large-scale are of great value to warrant these findings.

## Conclusion

Our results demonstrate that the serum BDNF levels were negatively associated with depressive symptoms in young Chinese adults with acne vulgaris. These findings suggest BDNF as a promising non-invasive biomarker in screening of depression in young adults’ patients with acne vulgaris. Definitely, large-scale cohort studies should be implemented to warrant the screening value of BDNF in clinical purpose.

## Additional file

Additional file 1: **Table S1.** Assessment of BDNF levels with age, disease durations, GAGS score, DLQI score, serum IL-6 and TNF-α levels. (DOCX 15 kb)

## Data Availability

The data is usable for this study only.

## Abbreviations

AIS: Athens insomnia scale
BDNF: Brain derived neurophic factor
DLQI: Dermatology life quality index
DSM-IV: Diagnostic and Statistical Manual of Mental Disorders-IV
GAGS: Global acne grading system
IL-6: Interleukin- 6
MMD: Major depressive disorder
PHQ-9: 9-item patient health questionnaire
ROC: Receiver operating characteristic curves
TNF-α: Tumor necrosis factor-alpha
