# Supplementary material for: Association of brain-derived neurotrophic factor levels and depressive symptoms in young adults with acne vulgaris
Source: BMC Psychiatry. 2019 Jun 24;19:193. doi: 10.1186/s12888-019-2182-8 (PMC6591989; doi:10.1186/s12888-019-2182-8)
Supplement: Supplementary file 1 — Table S1. Assessment of BDNF levels with age, disease durations, GAGS score, DLQI score, serum IL-6 and TNF-α levels. (DOCX 15 kb) [file 12888_2019_2182_MOESM1_ESM.docx]

**SUPPLEMENTAL MATERIAL**

**Association of brain-derived neurotrophic factor levels and depressive symptoms in young adults with acne vulgaris**

**Inventory of Supplemental Information**

**- Supplemental Table S1‎ (Page 1)**

**Table S1.**

**Table S1.** Assessment of BDNF levels with age, disease durations, GAGS score, DLQI score, serum IL-6 and TNF-α levels.

|  | **BDNF (ng/ml)** | |
| --- | --- | --- |
|  | ***r*** | ***P-value* ^a^** |
| Age (yrs.) | - 0.103 | 0.265 |
| Disease durations (yrs.) | - 0.102 | 0.270 |
| GAGS score | - 0.002 | 0.982 |
| DLQI score | - 0.015 | 0.868 |
| IL-6 (pg/ml) | 0.079 | 0.396 |
| TNF-α (pg/ml) | - 0.023 | 0.804 |
| All data expressed as mean ± SD (range) of the mean of individual groups.  ^a^ Spearman’s Rank correlation was used.  Abbreviation: PHQ-9, 9-item Patient Health Questionnaire; AIS, Athens Insomnia Scale; IL-6, interleukin-6; TNF-α, tumor necrosis factor-alpha. | | |
